# Supplementary material for: Nano-metal diborides-supported anode catalyst with strongly coupled TaOx/IrO2 catalytic layer for low-iridium-loading proton exchange membrane electrolyzer
Source: Nat Commun. 2023 Aug 23;14:5119. doi: 10.1038/s41467-023-40912-8 (PMC10447464; doi:10.1038/s41467-023-40912-8)
Supplement: Supplementary file 1 — Supplementary Information [file 41467_2023_40912_MOESM1_ESM.pdf]

Supplementary Information (SI) for:

Nano-metal diborides-supported anode catalyst with strongly coupled TaO_x/IrO_x catalytic layer for low-iridium-loading proton exchange membrane electrolyzer

Yuannan Wang,[1] Mingcheng Zhang,[1] Zhenye Kang,[2] Lei Shi,[1] Yucheng Shen,[1] Boyuan Tian,[3] Yongcun Zou,[1] Hui Chen[1,*] and Xiaoxin Zou[1,*]

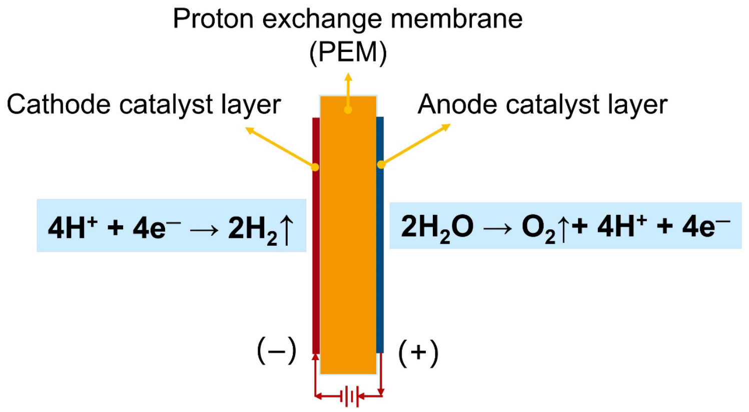

Supplementary Figure 1. Schematic diagram of catalyst coated membrane (CCM) in PEMWE.

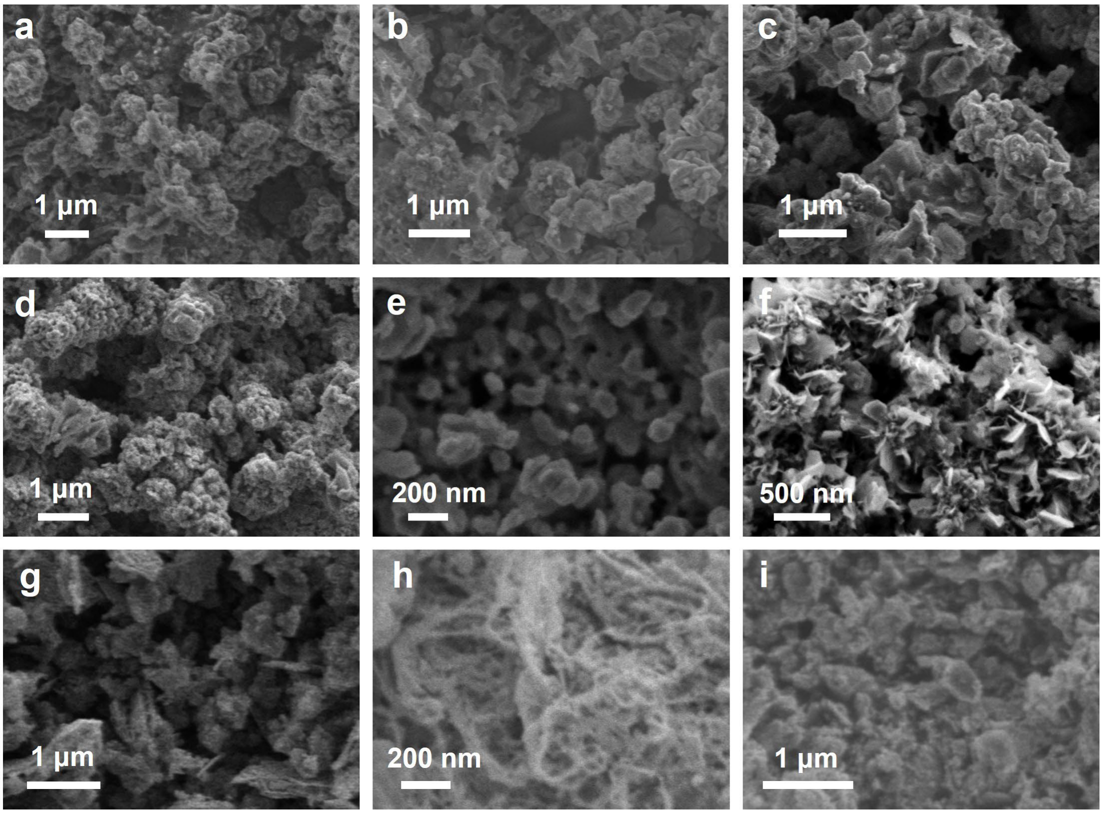

Supplementary Figure 2. SEM images of (a) TiB_2, (b) ZrB_2, (c) HfB_2, (d) VB_2, (e) NbB_2, (f) TaB_2, (g) MoB_2, (h) WB_2 and (i) ReB_2.

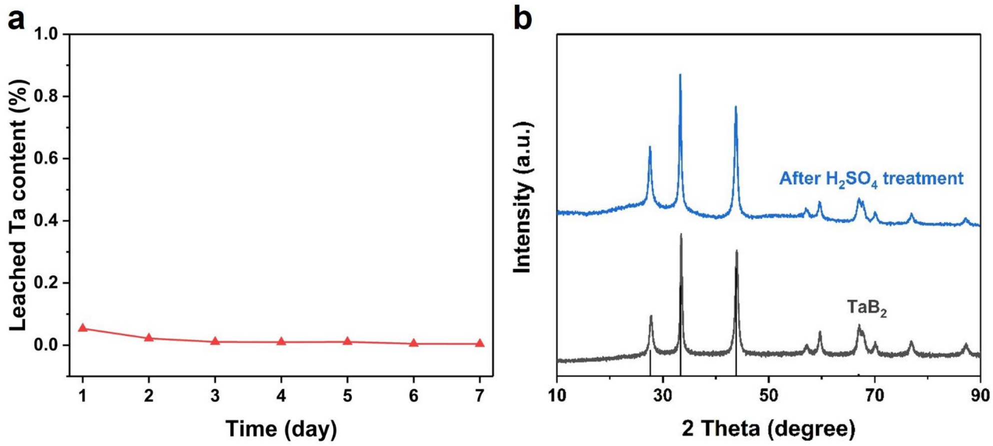

Supplementary Figure 3. (a) Content of leached Ta after the exposure of TaB_2 to 0.5 M H_2SO_4 at 80 ºC for 7 days, and (b) XRD patterns for TaB_2 before and after H_2SO_4 treatment.

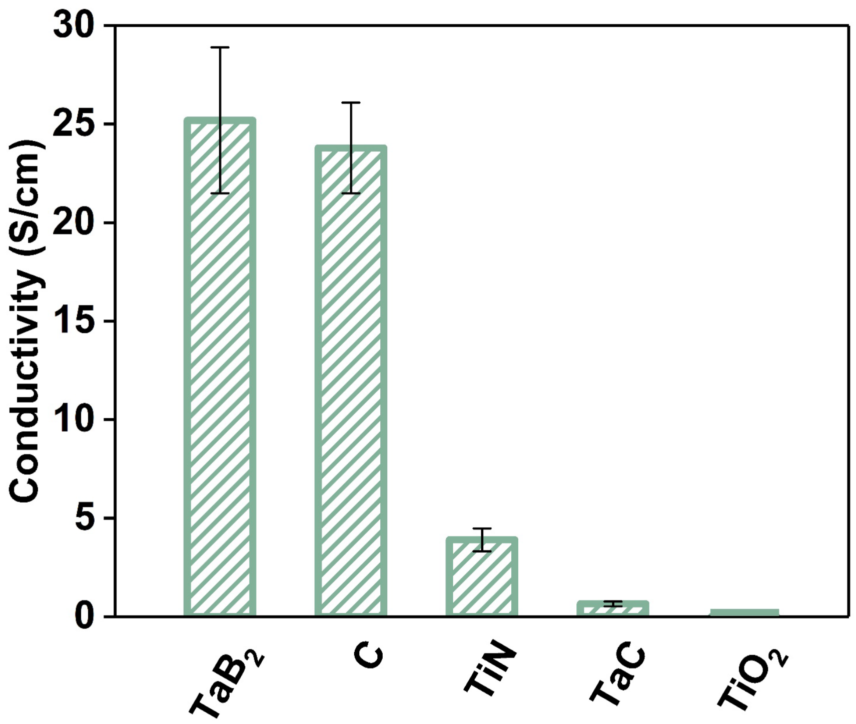

Supplementary Figure 4. Powder conductivity of TaB_2 and some other common support materials (*e.g.,* C, TiN, TaC, TiO_2) of electrocatalysts. The error bars represent standard deviations based on three measurements.

The conductivity of the powder sample was measured using a ST-2722 semiconductor resistivity of the powder tester, China. The powder sample was pressed into a tablet at the pressure of 25 MPa, and then the conductivity of the sample was obtained by four-point probe method.

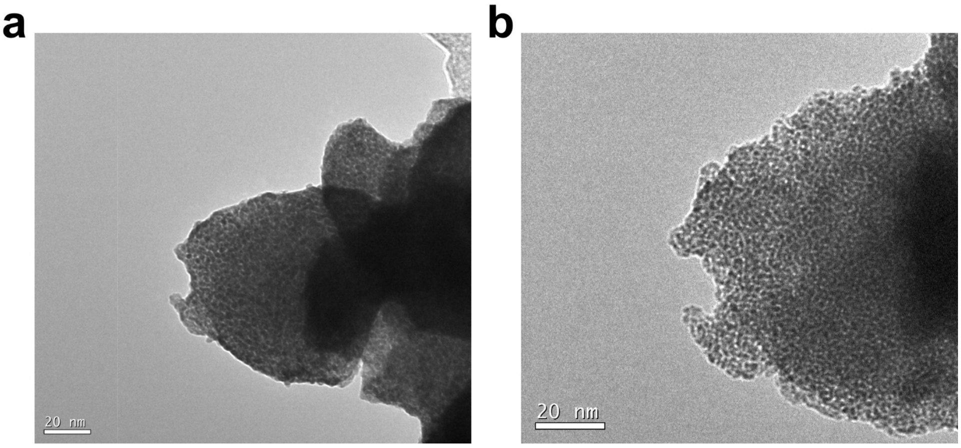

Supplementary Figure 5. TEM images of IrO_2 synthesized in molten $NaNO_3$.

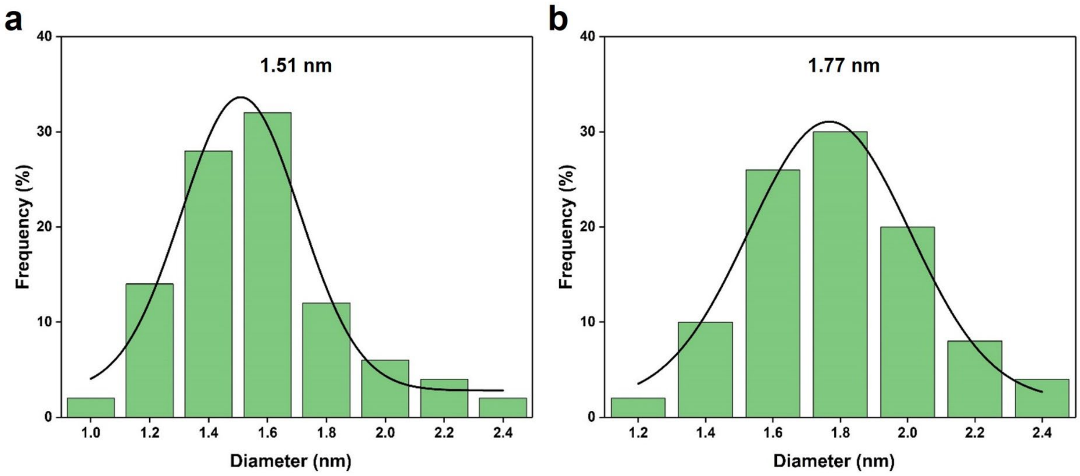

Supplementary Figure 6. Particle size distribution of (a) IrO_2@TaB_2 and (b) IrO_2.

The average IrO_2 particle size was obtained by measuring the size of 50 different particles from TEM images using the commercial software package Image-Pro Plus.

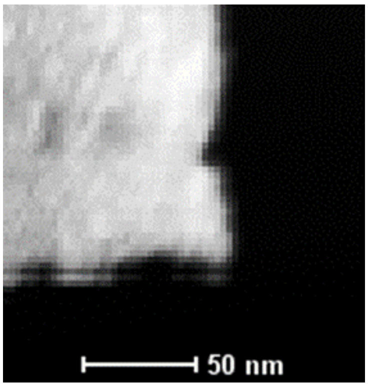

Supplementary Figure 7. STEM-HAADF image of $IrO_2@TaB_2$.

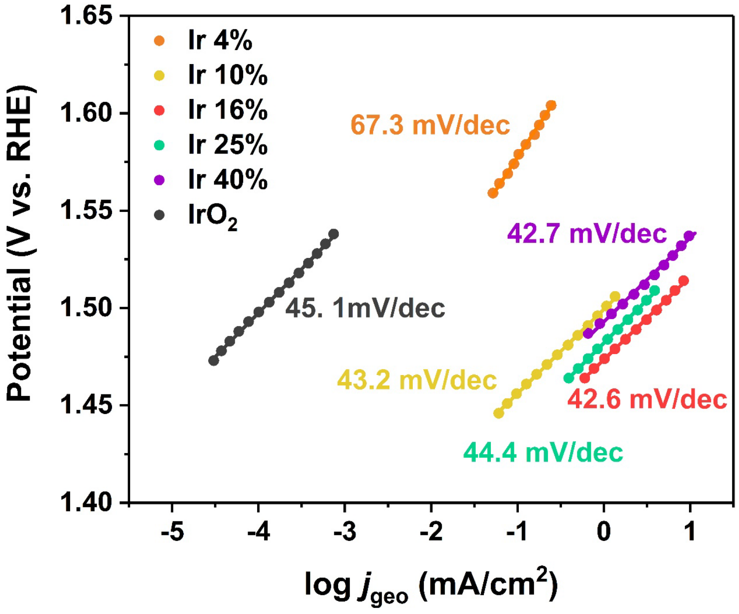

Supplementary Figure 8. Tafel plots for OER over IrO_2 and $IrO_2@TaB_2$ with different Ir contents.

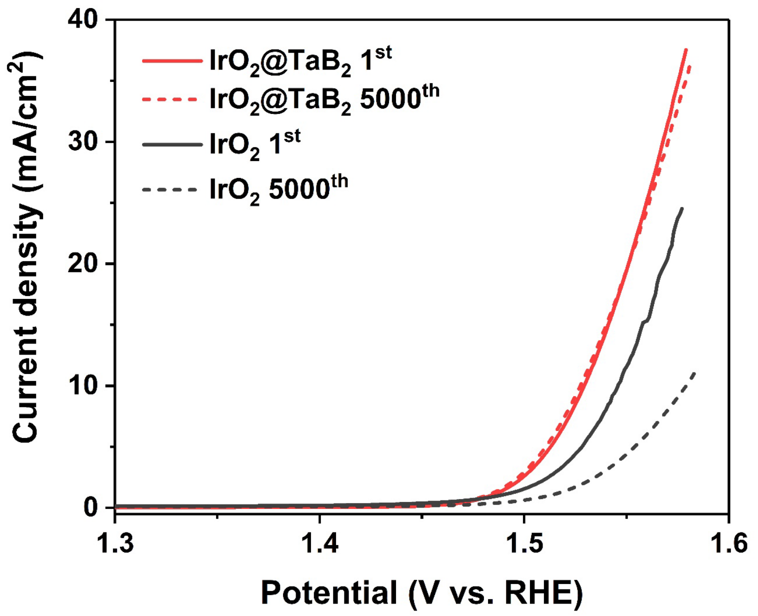

Supplementary Figure 9 The polarization curve for OER obtained from IrO_2@TaB_2 and IrO_2 before and after 5000 cycles.

The 5000 CV cycles were performed in a potential window of 1.2-1.6 V vs. RHE at a scan rate of 100 mV/s.

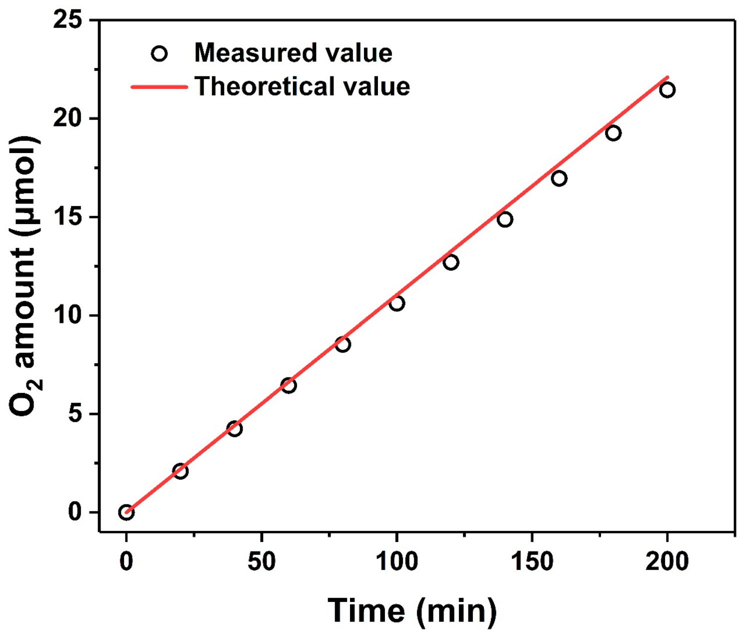

Supplementary Figure 10. The amount of O_2 theoretically calculated and experimentally measured during OER over IrO_2@TaB_2 catalyst versus time at a current density of 10 mA/cm^2.

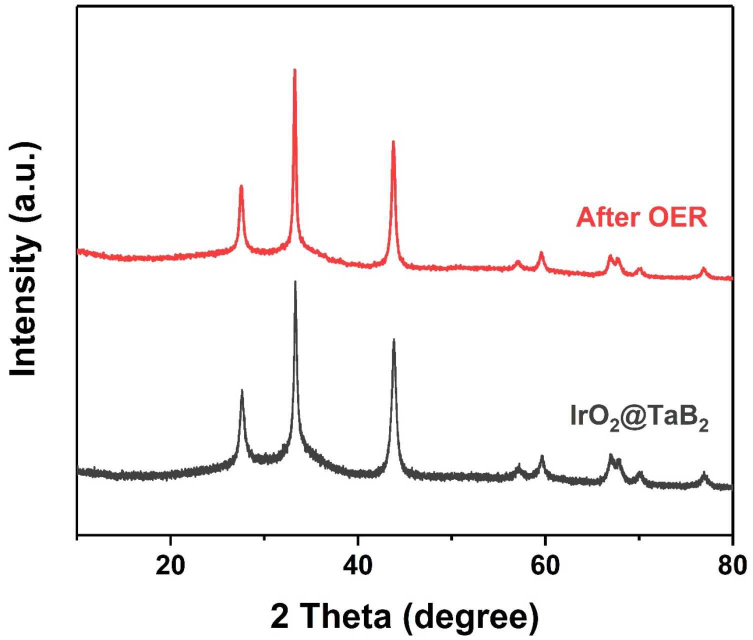

Supplementary Figure 11. Comparison of XRD patterns for IrO_2@TaB_2 before and after OER catalysis.

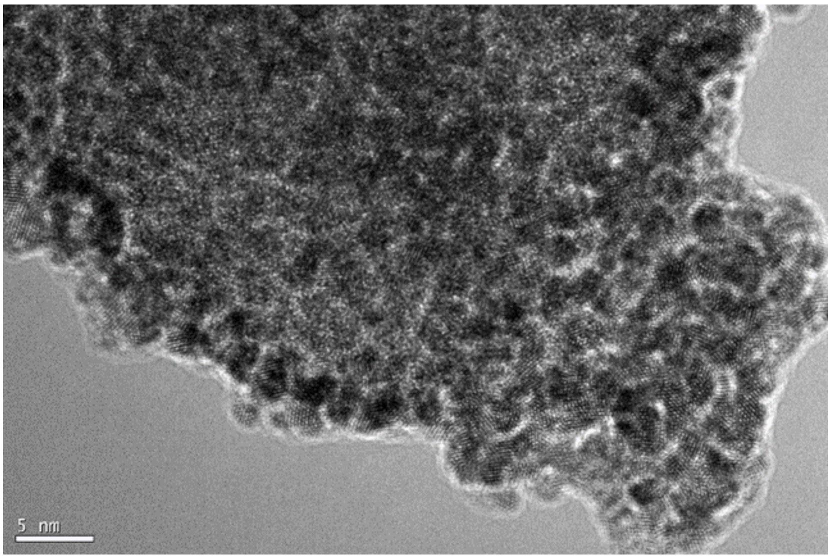

Supplementary Figure 12. TEM image of IrO_2@TaB_2 after OER catalysis.

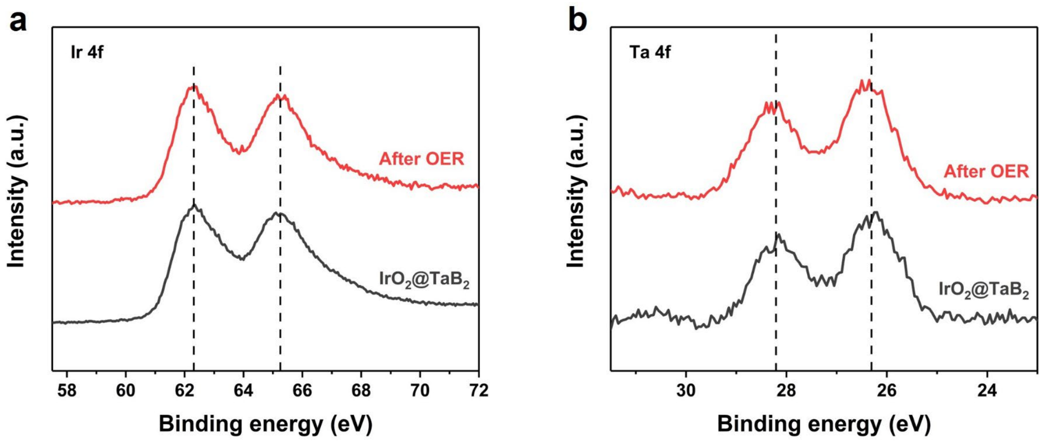

Supplementary Figure 13. Comparison of (a) Ir 4f and (b) Ta 4f XPS spectra for IrO_2@TaB_2 before and after OER catalysis.

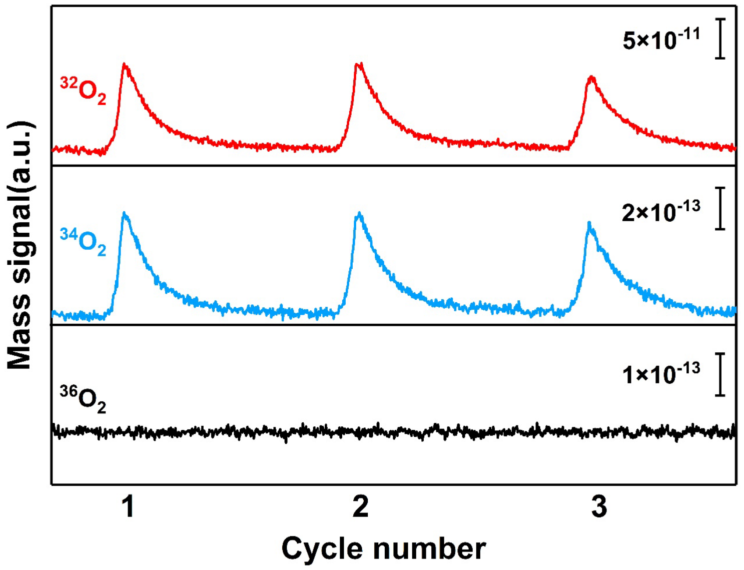

Supplementary Figure 14. DEMS signals of oxygen products for ^{18}O-labeled IrO_2 in 0.1 M $HClO_4$ aqueous.

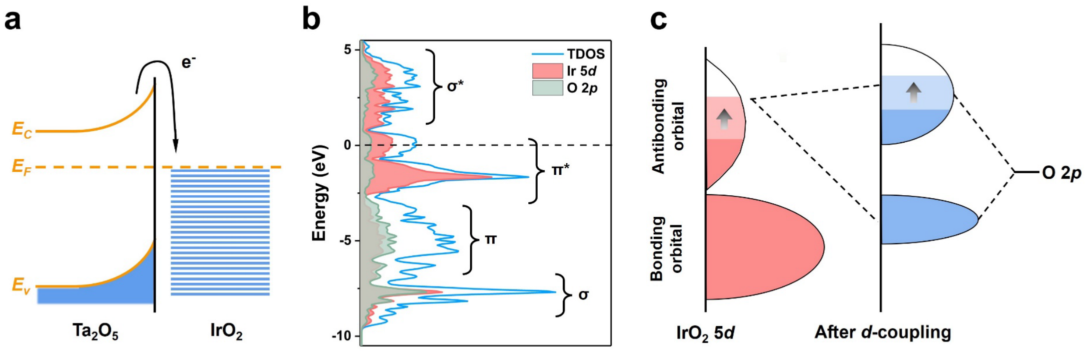

Supplementary Figure 15. (d) The Electron transfer in the interface of IrO_2-TaO_x heterojunction. (e) Projected density of state of IrO_2. (f) Schematic energy diagram for the binding of IrO_2 with oxygen.

IrO_2 is a highly conducting and metallic-like oxide with a work function of 4.2-4.5 eV,[1-3] while typical tantalum oxide (*i.e.*, Ta_2O_5) is a n-type semiconductor with a wide band gap of 4.4 eV and a work function of 3.2-4.2 eV.[4-7] That is say, the electrons in IrO_2 have a lower energy level than those in Ta_2O_5. After contact, a metal−semiconductor heterojunction is constructed between Ta_2O_5 and IrO_2, resulting in the formation of surface electric field and electronic interaction (**Supplementary Figure 15a**). The electrons will flow from conduction band of Ta_2O_5 to IrO_2 until the Fermi levels of Ta_2O_5 and IrO_2 reach equilibrium, leading to electron-rich IrO_2.

We further analyzed the electronic structure of IrO_2 by the density projected density of states (pDOS).[8] As shown in **Supplementary Figure 15b**, the hybridization of Ir 5d band with O 2p band forms bonding and antibonding orbitals. While the bonding orbitals composed of Ir 5d and O 2p bands lie below the Fermi level (between −10 and -2.5 eV), the antibonding orbitals mainly dominated by Ir 5d bands cross the Fermi level (between −2.5 and 5 eV). The largely empty antibonding state of IrO_2 will exhibit strong binding affinity to adsorbate. In fact, previous theoretical works have confirmed that the Ir sites in IrO_2 bind the oxygen intermediate too strongly such that the OER activity of IrO_2 is non-optimal on the OER volcano plot.[9-11] When IrO_2-TaO_x heterojunction is constructed, the antibonding states of IrO_2 are more fully occupied by electrons, which lowers surface oxygen adsorption of IrO_2 and consequently boosts the OER activity (**Supplementary Figure 15c**).

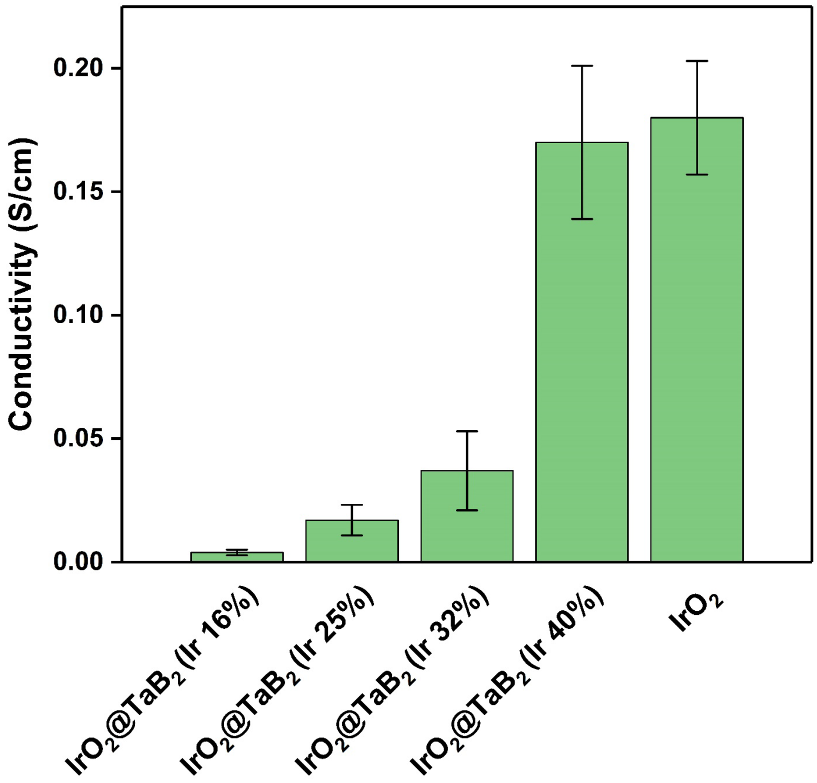

Supplementary Figure 16. Powder conductivity of IrO_2 and IrO_2@TaB_2 with different Ir content. The error bars represent standard deviations based on three measurements.

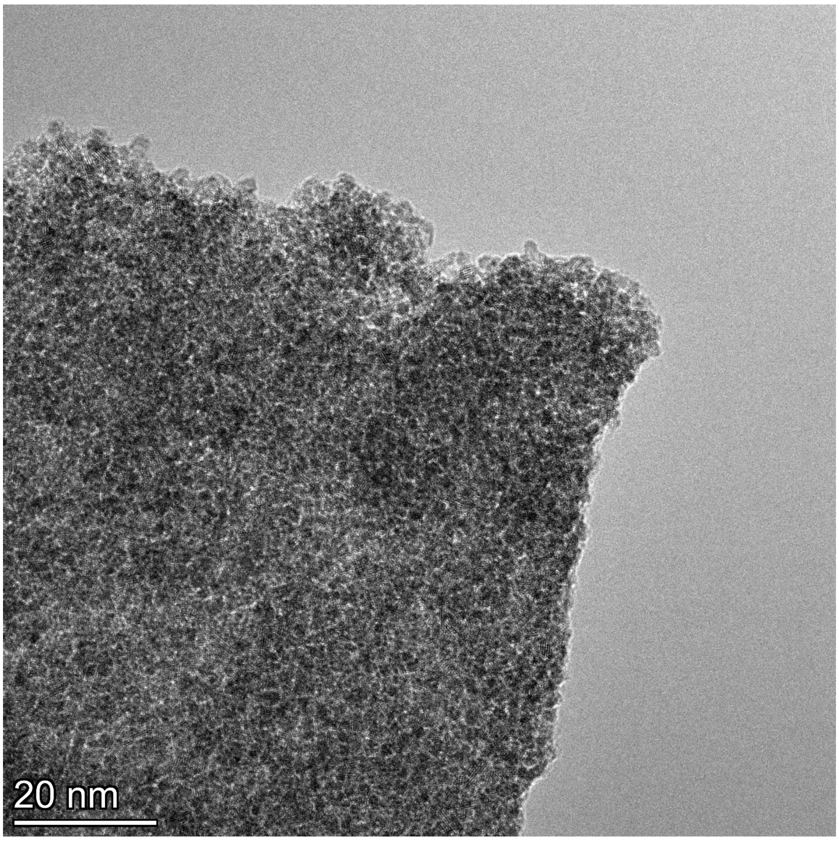

Supplementary Figure 17. TEM image of 40% IrO_2@TaB_2.

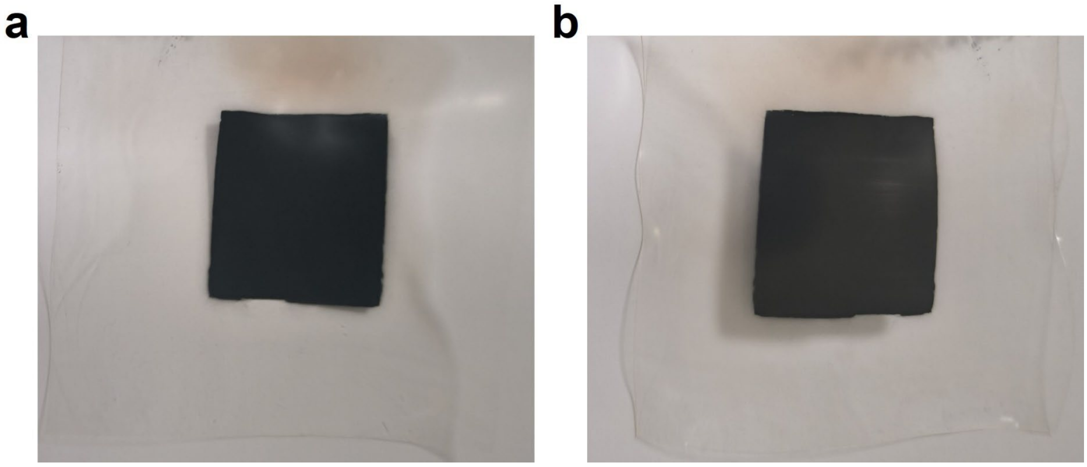

Supplementary Figure 18. Optical photographs of CCM with a 5 cm^2 working area, including (a) 40 % IrO_2@TaB_2 as anode catalyst on the N115 membrane, (b) 40% Pt/C as cathode catalyst on the N115 membrane.

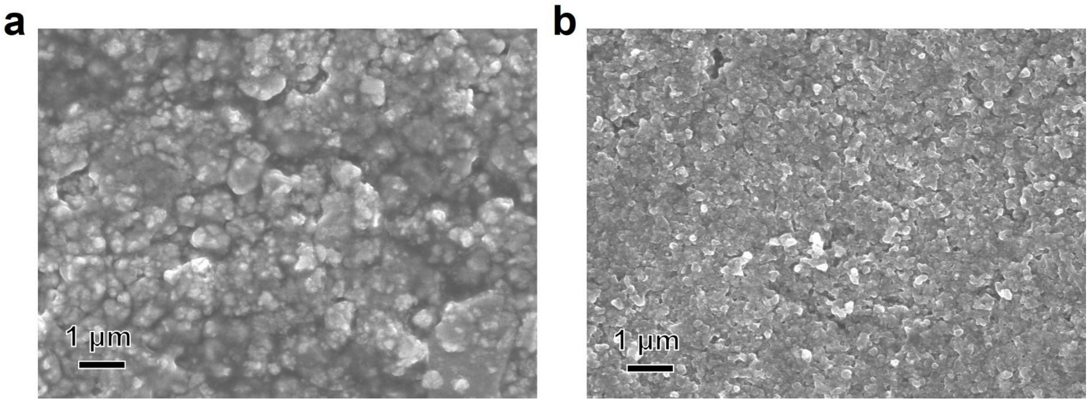

Supplementary Figure 19. SEM images of (a) IrO_2@TaB_2 as anode catalyst coated on the N115 membrane and (b) Pt/C as cathode catalyst coated on the N115 membrane.

As shown in **Supplementary Figure 19,** both the catalyst particles of IrO_2@TaB_2 anode and Pt/C cathode are composed of uniformly distributed agglomerates on the membrane surface.

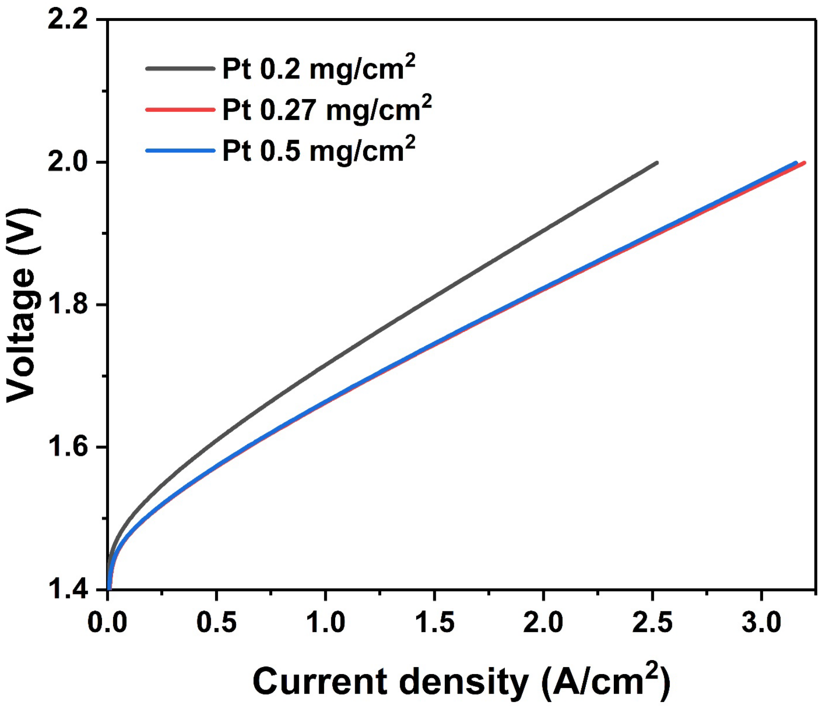

Supplementary Figure 20. Polarization curves of PEMWE using different content (0.2-0.5 mg cm^{-2}) of Pt at the cathode, while using IrO_2@TaB_2 anode under the same Ir loading of 0.15 mg_{Ir} cm^{-2}.

We consider 0.27 mg cm^{-2} as the optimized Pt loading at the cathode. When Pt content is increased to 0.5 mg cm^{-2}, the performance of PEMWE is not enhanced further. When Pt content is decreased to 0.2 mg cm^{-2}, the performance of PEMWE is decreased.

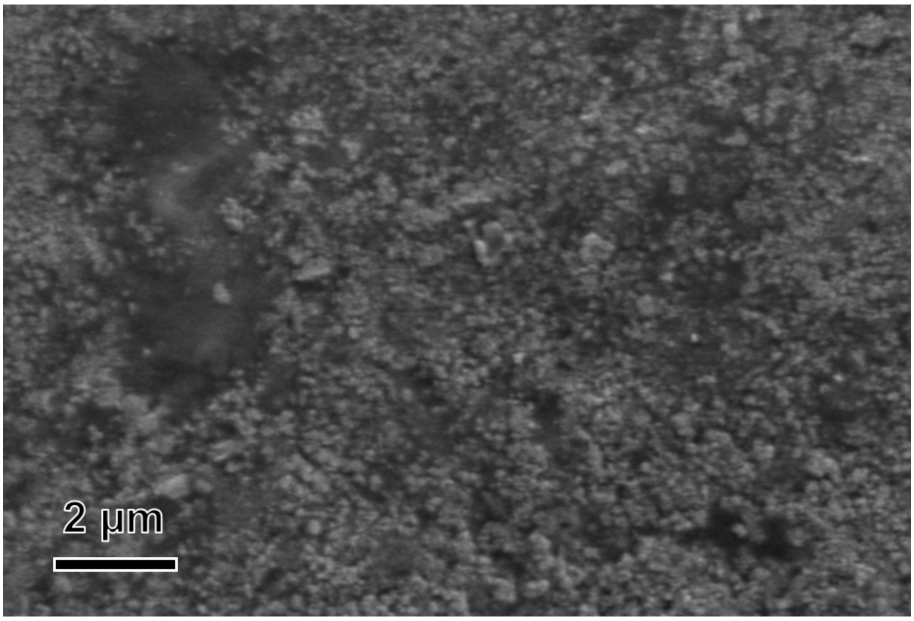

Supplementary Figure 21. SEM image of IrO_2 as anode catalyst (0.2 mg cm^{-2}) coated on the N115 membrane.

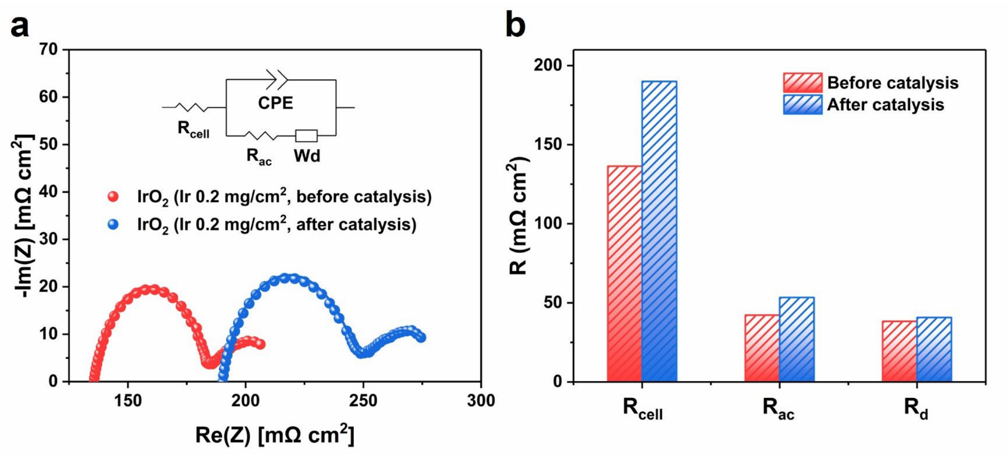

Supplementary Figure 22. (a) EIS curves of PEMWE using 0.2 mg cm^{-2} pure IrO_2 anode before and after electrochemical testing. The inset shows EEC model for EIS fitting. (b) The comparison of ohmic resistance, activation resistance, and diffusion resistance for PEMWE using 0.2 mg cm^{-2} pure IrO_2 anode before and after electrochemical testing.

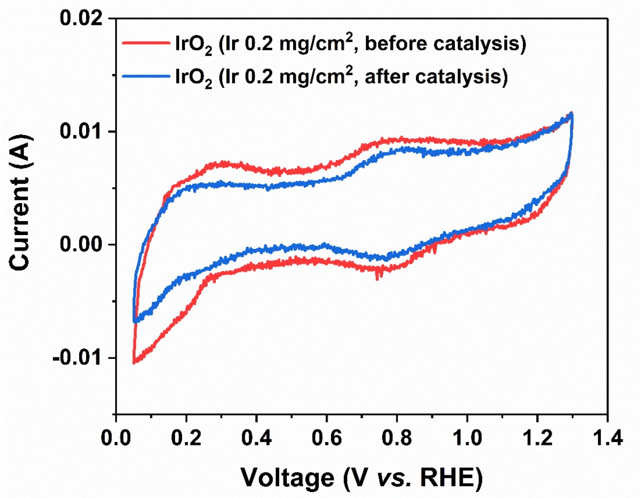

Supplementary Figure 23. The cyclic voltammograms of PEMWE using 0.2 mg cm^{-2} pure IrO_2 anode before and after electrochemical testing (0.05-1.3 V *vs*. RHE, 36 mV/s).

In order to investigate degradation processes of PEMWE using 0.2 mg cm^{-2} pure IrO_2 anode, we carry out chemical impedance spectroscopy (EIS) experiment after electrochemical testing, and fit the EIS Nyquist plots by equivalent electrical circuit (EEC) model (**Supplementary Figure 22**). The increase of the cell voltage was mainly due to large increases of the ohmic resistance and activation resistance. The ohmic resistance of the cell increases from 136 mΩ cm^2 to 190 mΩ cm^2, and the activation resistance increases from 42 mΩ cm^2 to 53 mΩ cm^2. The increased activation resistance also can be further supported by comparing the cyclic voltammograms before and after electrochemical testing of PEMWE using 0.2 mg cm^{-2} pure IrO_2 anode. As shown in **Supplementary Figure 23,** the number of electrocatalytic active sites decreases after electrochemical testing. These results indicate that unfavorable microstructural

evolutions (*e.g.*, agglomeration of catalyst particles, exfoliation of the catalyst layer) are obvious for low loading IrO_2 layer, leading to the reduction of electrocatalytic active sites and the increase of the ohmic resistance.

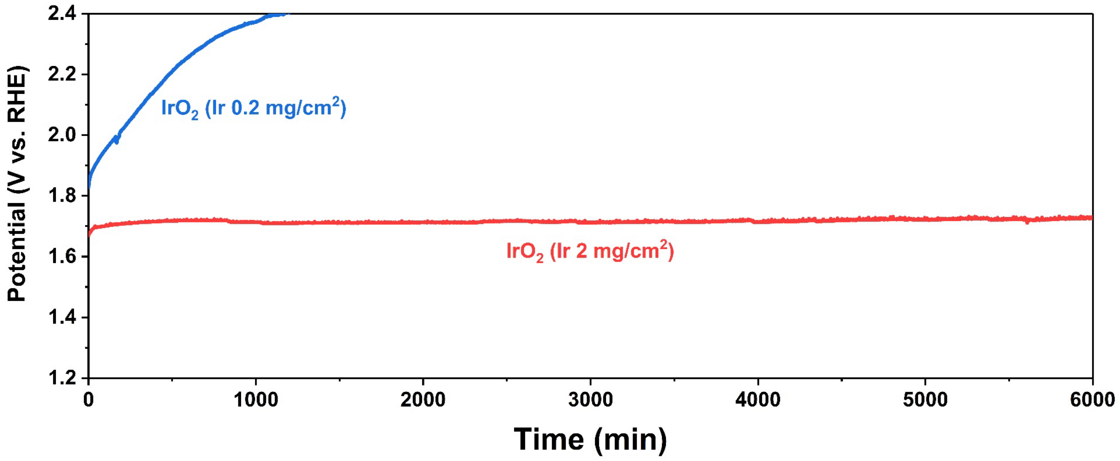

Supplementary Figure 24. Chronopotentiometry curve of PEMWE using IrO_2 anodes operated at 1 A cm^{-2} under the Ir loading of 0.2 and 2 mg_{Ir} cm^{-2}.

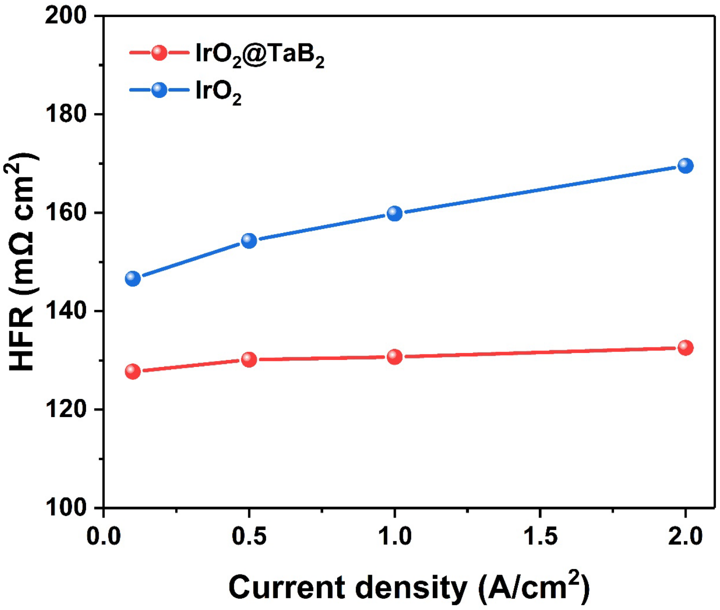

Supplementary Figure 25. The high frequency resistance (HFR) of $IrO_2@TaB_2$ and IrO_2.

Supplementary Table 1. Corresponding experimental parameters and chemical reagents used in the synthesis of metal diborides by metal disulfide.

Metal Diborides	Mass of metal disulfides (g)	Mass of boron (g)	Mass of molten salt (g)	Temperature (K)	Time (min)
TiB_2	0.112	0.074	4.2	1273	10
ZrB_2	0.155	0.074	4.2	1373	30
HfB_2	0.121	0.037	2.1	1373	30
VB_2	0.115	0.074	4.2	1273	10
NbB_2	0.157	0.074	4.2	1273	30
TaB_2	0.123	0.037	2.1	1273	10
MoB_2	0.160	0.074	4.2	1273	10
WB_2	0.124	0.037	2.1	1373	30
ReB_2	0.125	0.037	2.1	1173	10

Supplementary Table 2. The energy calculation results of metal disulfide and corresponding metal diborides.

**Structure**	**Energy per molecule / eV**	**Structure**	**Energy per molecule / eV**	**ΔH/ (kJ mol^{-1})**
HfS_2	-22.93	HfB_2	-26.39	334.6
MoS_2	-21.85	MoB_2	-25.21	343.6
NbS_2	-21.64	NbB_2	-25.83	264.1
TaS_2	-23.15	TaB_2	-27.16	281.7
TiS_2	-19.95	TiB_2	-24.42	237.5
VS_2	-19.81	VB_2	-24.62	204.7
WS_2	-23.67	WB_2	-27.37	314.0
ZrS_2	-21.44	ZrB_2	-24.97	328.0
ReS_2	-22.37	ReB_2	-27.07	216.7

Supplementary Table 3. The BET surface areas and leached metal contents of nine metal diborides.

Sample	BET Surface Area (m^2 g^{-1})	Leached metal content (%)[a]
TiB_2	45.8	14.6
ZrB_2	43.3	52.6
HfB_2	23.0	83.3
VB_2	16.6	65.0
NbB_2	27.0	0.1
**TaB_2**	**54.6**	**0.04**
MoB_2	40.8	12.9
WB_2	54.5	13.7
ReB_2	8.6	19.1

[a] The metal diborides were exposed to 0.5 M H_2SO_4 at 80°C for 24 h and the leached metal content was obtained by ICP-OES test.

Supplementary Table 4. The crust abundance and price of Ir and Ta.

Metal	Abundance of chemical elements in Earth's crust (ppm)[a]	Metal price (USD/kg)[b]
Ir	0.000003	164662.0
**Ta**	**2**	**367.5**

[a] The abundance is obtained from: https://environmentalchemistry.com/yogi/periodic/.
[b] The metal price is obtained from: https://www.metal.com/price.

Supplementary Table 5. EXAFS fitting parameters at the Ir L1-edge for IrO_2 and IrO_2@TaB_2 (S_0^2=0.900).

Sample	Shell	CN^a	R(Å)^b	σ^2(Å^2)^c	ΔE_0(eV)^d	R factor
IrO_2	Ir-O	6.0	2.00	0.0037	-1.8	0.0094
	Ir-Ir	5.3	3.08	0.0053		
	Ir-O	5.9	3.15	0.0037		
	Ir-Ir	6.6	3.59	0.0053		
IrO_2@TaB_2	Ir-O	6.0	2.04	0.0037	1.1	0.0180
	Ir-Ir	6.7	3.08	0.0061		
	Ir-O	5.1	3.24	0.0037		
	Ir-Ir/Ta	3.4	3.62	0.0061		

^{a}*CN*, coordination number; ^{b}*R*, distance between absorber and backscatter atoms; $^c\sigma^2$, Debye-Waller factor to account for both thermal and structural disorders; $^d\Delta E_0$, inner potential correction; *R* factor indicates the goodness of the fit. Error bounds (accuracies) that characterize the structural parameters obtained by EXAFS spectroscopy were estimated as N ± 20%; R ± 1%; σ^2 ± 20%; ΔE_0 ± 20%. $S0^2$ was fixed to 0.900. Fitting range: $3.0 \leq k$ (/Å) ≤ 11.8 and $1.0 \leq R$ (Å) ≤ 4.0 (IrO_2 and Ir). A reasonable range of EXAFS fitting parameters: $0.700 < S_0^2 < 1.000$; $CN > 0$; $\sigma^2 > 0$ Å^2; $\Delta E_0 < 10$ eV; R factor < 0.02.

Supplementary Table 6. The ECSAs of IrO_2 and IrO_2@TaB_2 with different Ir content.

Sample	IrO_2@TaB_2 (Ir 4%)	IrO_2@TaB_2 (Ir 10%)	IrO_2@TaB_2 (Ir 16%)	IrO_2@TaB_2 (Ir 25%)	IrO_2@TaB_2 (Ir 40%)	IrO_2
m_{Ir} (mg) on working electrode	0.0008	0.002	0.0032	0.005	0.008	0.016
ECSA (cm^2)	0.59	5.6	17.3	16.9	31.1	44.5
ECSA normalized by Ir mass ($m^2\ g_{Ir}^{-1}$)	73.8	280	541	338	389	278

The Ir mass-normalized ECSA values become larger with increasing Ir content in IrO_2@TaB_2, and reach its maximum for the 16 wt% IrO_2@TaB_2 sample (541 $m^2\ g_{Ir}^{-1}$).

Supplementary Table 7 The comparison of iridium mass activities for OER of IrO_2@TaB_2 with some representative Ir-based electrocatalysts in acidic media.

Catalysis	Electrolyte	Iridium content (%)	j_{Ir} (A/g_{Ir}@1.53 V)	Ref.
IrO_2@TaB_2	0.1 M $HClO_4$	16	345	This work
IrO_2	0.1 M $HClO_4$	84	33	This work
IrO_2@ Ir/TiN	0.5 M H_2SO_4	60	100	12
IrO_2-TiO_2	0.1 M $HClO_4$	55	70	13
$Pr_2Ir_2O_7$	0.1 M $HClO_4$	50	424.5	14
$IrO_2/Nb_{0.2}Ti_{0.8}O_2$	0.1 M $HClO_4$	50	80	15
6H-$SrIrO_3$	0.5 M H_2SO_4,	58.6	110	16
3C-$SrIrO_3$	0.5 M H_2SO_4	58.6	50	16
Li-IrO_x	0.1 M $HClO_4$	84	140	17
IrO_x-ATO	0.05 M H_2SO_4	20	200	18
$K_{0.25}IrO_2$	0.1 M $HClO_4$	82.1	19	19
Sr_2IrO_4	0.1 M $HClO_4$	44.6	280	20
Ca_2IrO_4	0.1 M $HClO_4$	57.1	44	21
$Ir/W_xTi_{1-x}O_2$	0.1 M $HClO_4$	38	139	22

Supplementary Table 8 Reported performances of PEMWE anode catalysts and corresponding parameters.

Catalysis	Electrolyte	Membrane	Activity (A cm^{-2}@1.9 V)	PGM loading (mg cm^{-2})	Ref.
IrO_2@TaB_2	H_2O	N115	2.52	0.42	This work
$Ta_{0.1}Tm_{0.1}Ir_{0.8}O_{2-\delta}$	0.5 M H_2SO_4	N117	1.8	0.24	23
Ir/Nb_2O_{5-x}	H_2O	N115	3.2	2.2	24
Ni-RuO_2	0.1 M $HClO_4$	N117	0.95	2.5	25
Ir/B_4C	H_2O	N115	2.6	1	26
Ir@WO_xNRs	H_2O	N115	1.81	0.9	27
IrO_2/ATO	H_2O	N115	1.7	2.5	28
$IrO_2/Ti_{1-x}W_xO_2$	H_2O	N117	1.3	2.64	29
Sr_2CaIrO_6	H_2O	NR212	2.45	0.8	30
$W_{0.7}Ir_{0.3}O_y$	0.1 M $HClO_4$	NR212	0.61	0.65	31
Ir-ND/ATO	H_2O	NR212	1.9	1.4	32
IrO_x NP/NF	H_2O	N115	2.51	0.7	33
Porous IrO_x	H_2O	N115	2.3	2.2	34
Porous $Ir_{0.7}Ru_{0.3}O_2$	H_2O	N115	2.2	2	34
IrO_2@TiN_{1+x}	H_2O	N117	1.95	1.6	1

References for SI

1 Wang, S. *et al.* Defects tailoring IrO_2@TiN_{1+x} nano-heterojunctions for superior water oxidation activity and stability. *Mater. Chem. Front.* **5**, 8047-8055 (2021).

2 Ping, Y., Goddard, W. A., III & Galli, G. A. Energetics and Solvation Effects at the Photoanode/Catalyst Interface: Ohmic Contact versus Schottky Barrier. *J. Am. Chem. Soc.* **137**, 5264-5267 (2015).

3 Yang, Y. *et al.* IrO_x@In_2O_3 Heterojunction from Individually Crystallized Oxides for Weak-Light-Promoted Electrocatalytic Water Oxidation. *Angew. Chem. Int. Ed.* **60**, 26790-26797 (2021).

4 Blonkowski, S., Regache, M. & Halimaoui, A. Investigation and modeling of the electrical properties of metal–oxide–metal structures formed from chemical vapor deposited Ta_2O_5 films. *J. Appl. Phys.* **90**, 1501-1508 (2001).

5 Rasheed, U. *et al.* Resistive switching characteristics and theoretical simulation of a Pt/a-Ta_2O_5/TiN synaptic device for neuromorphic applications. *J. Alloy. Compd.* **877**, 160204 (2021).

6 Zhang, H., Zhou, Z., Yang, B. & Gao, M. The Influence of Carboxyl Groups on the Photoluminescence of Mercaptocarboxylic Acid-Stabilized CdTe Nanoparticles. *J. Phys. Chem. B* **107**, 8-13 (2003).

7 Wan, Y. *et al.* Tantalum Oxide Electron-Selective Heterocontacts for Silicon Photovoltaics and Photoelectrochemical Water Reduction. *ACS Energy Lett.* **3**, 125-131 (2018).

8 Ping, Y., Galli, G. & Goddard, W. A., III. Electronic Structure of IrO_2: The Role of the Metal d Orbitals. *J. Phys. Chem. C* **119**, 11570-11577 (2015).

9 Man, I. C. *et al.* Universality in Oxygen Evolution Electrocatalysis on Oxide Surfaces. *ChemCatChem* **3**, 1159-1165 (2011).

10 Chen, H. *et al.* Optimization of Active Sites via Crystal Phase, Composition, and Morphology for Efficient Low-Iridium Oxygen Evolution Catalysts. *Angew. Chem. Int. Ed.* **59**, 19654-19658 (2020).

11 Chen, H. *et al.* Protonated Iridate Nanosheets with a Highly Active and Stable Layered Perovskite Framework for Acidic Oxygen Evolution. *ACS Catal.* **12**, 8658-8666 (2022).

12 Li, G. Q. *et al.* Boosted Performance of Ir Species by Employing TiN as the Support toward Oxygen Evolution Reaction. *ACS Appl. Mater. Interfaces* **10**, 38117-38124 (2018).

13 Oakton, E. *et al.* IrO_2-TiO_2: A High-Surface-Area, Active, and Stable Electrocatalyst for the Oxygen Evolution Reaction. *ACS Catal.* **7**, 2346-2352 (2017).

14 Shang, C. Y. *et al.* Electron Correlations Engineer Catalytic Activity of Pyrochlore Iridates for Acidic Water Oxidation. *Adv. Mater.* **31**, 1805104 (2019).

15 Hao, C. P., Lv, H., Mi, C. E., Song, Y. K. & Ma, J. X. Investigation of Mesoporous Niobium-Doped TiO_2 as an Oxygen Evolution Catalyst Support in an SPE Water Electrolyzer. *ACS Sustainable Chem. Eng.* **4**, 746-756 (2016).

16 Yang, L. *et al.* Efficient oxygen evolution electrocatalysis in acid by a perovskite with face-sharing IrO_6 octahedral dimers. *Nat. Commun.* **9**, 5236 (2018).

17 Gao, J. J. *et al.* Breaking Long-Range Order in Iridium Oxide by Alkali Ion for

Efficient Water Oxidation. *J. Am. Chem. Soc* **141**, 3014-3023 (2019).
18 Oh, H. S. *et al.* Electrochemical Catalyst-Support Effects and Their Stabilizing Role for IrO_x Nanoparticle Catalysts during the Oxygen Evolution Reaction. *J. Am. Chem. Soc* **138**, 12552-12563 (2016).
19 Sun, W., Song, Y., Gong, X. Q., Cao, L. M. & Yang, J. Hollandite Structure $K_{x\approx0.25}IrO_2$ Catalyst with Highly Efficient Oxygen Evolution Reaction. *ACS Appl. Mater. Interfaces* **8**, 820-826 (2016).
20 Zhang, R. H. *et al.* First Example of Protonation of Ruddlesden-Popper Sr_2IrO_4: A Route to Enhanced Water Oxidation Catalysts. *Chem. Mater.* **32**, 3499-3509 (2020).
21 Wu, Y. Y. *et al.* Highly Efficient Oxygen Evolution Activity of Ca_2IrO_4 in an Acidic Environment due to Its Crystal Configuration. *Acs Omega* **3**, 2902-2908 (2018).
22 Zhao, S., Stocks, A., Rasimick, B., More, K. & Xu, H. Highly Active, Durable Dispersed Iridium Nanocatalysts for PEM Water Electrolyzers. *J. Electrochem. Soc.* **165**, F82-F89 (2018).
23 Hao, S. Y. *et al.* Torsion strained iridium oxide for efficient acidic water oxidation in proton exchange membrane electrolyzers. *Nat. Nanotechnol.* **16**, 1371–1377 (2021).
24 Shi, Z. *et al.* Enhanced Acidic Water Oxidation by Dynamic Migration of Oxygen Species at the Ir/Nb_2O_{5-x} Catalyst/Support Interfaces. *Angew. Chem. Int. Ed.* **61**, e202212341 (2022).
25 Wu, Z.-Y. *et al.* Non-iridium-based electrocatalyst for durable acidic oxygen evolution reaction in proton exchange membrane water electrolysis. *Nat. Mater.* **22**, 100-108 (2023).
26 Islam, J. *et al.* Enhancing the activity and durability of iridium electrocatalyst supported on boron carbide by tuning the chemical state of iridium for oxygen evolution reaction. *J. Power Sources* **512**, 230506 (2021).
27 Jiang, G. *et al.* Low-Loading and Highly Stable Membrane Electrode Based on an $Ir@WO_xNR$ Ordered Array for PEM Water Electrolysis. *ACS Appl. Mater. Interfaces* **13**, 15073-15082 (2021).
28 Puthiyapura, V. K., Mamlouk, M., Pasupathi, S., Pollet, B. G. & Scott, K. Physical and electrochemical evaluation of ATO supported IrO_2 catalyst for proton exchange membrane water electrolyser. *J. Power Sources* **269**, 451-460 (2014).
29 Lv, H. *et al.* Synthesis and activities of $IrO_2/Ti_{1-x}W_xO_2$ electrocatalyst for oxygen evolution in solid polymer electrolyte water electrolyzer. *J Electroanal Chem* **833**, 471-479 (2019).
30 Retuerto, M. *et al.* Highly active and stable OER electrocatalysts derived from Sr_2MIrO_6 for proton exchange membrane water electrolyzers. *Nat. Commun.* **13**, 7935 (2022).
31 Yan, T. *et al.* IrO_2 Nanoparticle-Decorated Ir-Doped $W_{18}O_{49}$ Nanowires with High Mass Specific OER Activity for Proton Exchange Membrane Electrolysis. *ACS Appl. Mater. Interfaces* **15**, 6912-6922 (2023).
32 Oh, H. S., Nong, H. N., Reier, T., Gliech, M. & Strasser, P. Oxide-supported Ir nanodendrites with high activity and durability for the oxygen evolution reaction in acid PEM water electrolyzers. *Chem. Sci.* **6**, 3321-3328 (2015).

33 Hegge, F. *et al.* Efficient and Stable Low Iridium Loaded Anodes for PEM Water Electrolysis Made Possible by Nanofiber Interlayers. *ACS Appl. Energy Mater.* **3**, 8276-8284 (2020).
34 Faustini, M. *et al.* Hierarchically Structured Ultraporous Iridium-Based Materials: A Novel Catalyst Architecture for Proton Exchange Membrane Water Electrolyzers. *Adv. Energy Mater.* **9**, 1802136 (2019).
